# Mortality rate of high cardiovascular risk patients with mild cognitive impairment

**DOI:** 10.1038/s41598-022-15823-1

**Published:** 2022-07-13

**Authors:** Teodora Yaneva-Sirakova, Latchezar Traykov

**Affiliations:** 1 Department of Internal Medicine, Medical University Sofia, UMHAT “Alexandrovska” EAD, Cardiology Clinic, Georgi Sofiiski Str 1, 1431 Sofia, Bulgaria; 2 Department of Neurology, Medical University Sofia, UMHAT “Alexandrovska” EAD, Neurology Clinic, Bulgarian Academy of Sciences, Sofia, Bulgaria; 3 Present Address: Acibadem City Clinic Cardio-Vascular Center, Sofia, Bulgaria

**Keywords:** Cognitive ageing, Cognitive neuroscience

## Abstract

People with mild cognitive impairment (MCI) may be at higher risk of death than normal aging ones. On the other hand, patients with cardiovascular risk factors are also with higher risk of death. It may be logical to question then if the combination of MCI and cardio-vascular risk factors (in most cases arterial hypertension) can lead to higher mortality rate than expected both for high cardio-vascular risk patients and for the general population. This hypothesis is important in the light of effective early screening and prophylaxis. The general death rate of patients with very high-cardio-vascular-risk was compared in the subgroups of normal cognition and MCI. We used MMSE and MoCA (reassessment 6 months apart), Geriatric Depression scale and 4-point version of the scale for evaluating the performance in instrumental activities of daily living (4-IADL) in 249 patients. The patients also had laboratory testing, ambulatory blood pressure monitoring, ECG and echocardiography. The general mortality rate of this very high cardio-vascular risk group was assessed 8–10 years afterwards and also compared to the general national death rate published for the corresponding period from the National Social Security Institute of Bulgaria. We registered significantly higher general death rate in patients with MCI and very high cardio-vascular risk as compared to the group without MCI. The logistic regression analysis attributed approximately 14.6% of the mortality rate in this high-risk group to MCI. The major cardio-vascular risk factor was arterial hypertension—with 63.85% of the patients with home blood pressure values not in the target range at the initial cognitive screening. During the neuropsychological reevaluation 56.43% were with poor control despite the multidrug antihypertensive regimen. It is known that MCI is correlated with cardiovascular risk factors with the leading role of arterial hypertension. We found that the combination of MCI and arterial hypertension can lead to higher mortality rate than in the general aging population. This has important clinical implications for the everyday practice.

## Introduction

More than 55 million people worldwide are diagnosed currently with dementia, with annual diagnosis rate of 10 million^1^. That makes dementia the seventh cause of death, with very high and ever rising rate of years of life lost, lost to disability and disability-adjusted life years^2^. For Bulgaria—around 100,000 people (out of 5 millions) are currently diagnosed with dementia, with possibly very high covered morbidity^3^. Dementia is correlated with elevated risk of death^4^. Mild cognitive impairment is a bridging state between normal aging and dementia, and is characterised with cognitive impairment with minimal impairment of instrumental activities of daily living as a main difference from dementia, and very high age adjusted prevalence rate (6.7% for ages 60–64, 8.4% for ages 65–69, 10.1% for 70–74, 14.8% for 75–79, and 25.2% for 80–84 years), cumulative dementia incidence 14.9% and no proven pharmacological treatment^5^. There are some studies in the general population of aging adults, that show higher mortality in people with cognitive impairment without manifest dementia^6^. Population-based studies suggest high impact of education, exercise and heart disease on death rate in advance-aged people (70–89) with mild cognitive impairment (MCI)^7^. A Norwegian register showed a significant loss in life expectancy for patients with MCI and all dementia subtypes (diagnosis made with neuropsychological testing)^8^. Patients with cardiovascular risk factors (diabetes, obesity, smoking, arterial hypertension, dyslipidemia, male sex, age) are also with elevated risk of death^9^. This high cardio-vascular risk group is also at an elevated risk for transition from MCI to dementia^10^. The leading risk factor is arterial hypertension. According to NCD Risk Factor Collaboration (NCD-RisC)^11^ hypertension morbidity doubled in the last 20 years and despite the universal measures control rates remained insufficient in the developed countries (around 50%) and poor in the developing countries (around 25%). Hypertension is the leading cause of more than 8.5 million deaths from stroke, ischemic coronary artery disease, renal disease^12^. Hypertension is associated with higher risk of dementia and MCI in young adults^13^ and optimal blood pressure control before age of 70 was important for the reduction of cognitive impairment morbidity^14^, but not in the very elderly group^15,16^. Cardio-vascular risk factors, with the leading significance of arterial hypertension, modify the risk of death from dementia or MCI^17^. Daen et al.^18^ found an U-shaped age-dependent association between mortality rate form dementia and systolic blood pressure. The conclusion for the diastolic blood pressure was the same, but less distinct. Based on the current knowledge, cardio-vascular risk factors, and especially arterial hypertension, in midlife lead to morphological and functional changes in the brain associated with cognitive decline later in life. The major mechanisms involved may be: lacunar infarcts, strategic strokes, small vessel disease, impaired blood–brain barrier and neuro-vascular unit functioning, reduced beta- amyloid clearance^19^. A study form Mayo Clinic^20^ found that mortality in MCI patients varied with sex, education, history of heart disease, and physical activity. However, studies which make triple correlation between MCI, cardio-vascular risk factors and death rate are scarce.

## Hypothesis

The combination of MCI and hypertension (and other cardio-vascular risk factors as well) may lead to even higher mortality rate than the current baseline.

This finding would be important in the light of effective early screening and prophylaxis, for real patients in the every day practice, who are not with a single isolated disease or risk factor. Crude death rate of Bulgaria for 2013 year for example was 14.4 per 1000 and it was much higher than the specific for EU 13 and WHO European region. Similarly, the mortality profile for Bulgaria is with very high percent for leading major cause of death per 100,000 population for diseases of the circulatory system (198.6/100,000 for men and 69.7/100,000 for women aged below 64 years—above that age, the mortality rate is even higher)^21^. This cardio-vascular death rate is between 1.6 (for males) and 1.8 (for females) times higher than the average rate in the Eastern European Region.

## Goal

We aimed at analyzing if there was any difference in the mortality rate between the groups of high-cardio-vascular risk patients with and without MCI.

## Materials and methods

For the given period from 2009 to 2012 year 269 patients with arterial hypertension underwent screening for MCI and reevaluated minimum 6 months after the initial evaluation. For 249 of them we found mortality data from the national registries and only these were included in the current analysis after 8–10 years of follow-up. All of them were with arterial hypertension and the majority of them—with additional cardiovascular risk factors (high and very high 10 year mortality risk defined by SCORE). The group was studied twice during the initial screening period. The second evaluation was at least 6 months after the inclusion (initial) assessment. That was the first phase of the study. During that phase we studied the cognitive performance of patients with arterial hypertension and various other cardio-vascular risk factors by performing a clinical screening with neuropsychological tests. After the second reevaluation the mortality rate of the patients was followed for 8–10 years. The first and the second evaluation included full medical history, physical examination, laboratory testing, ECG, echocardiography, ambulatory blood pressure monitoring, neuropsychological testing. The survey was conducted by two professionals: a cardiologist and a neurologist–neuropsychologist. The mortality rate was assessed via the national data base(National Social Security Institute of Bulgaria) and was done in mid 2019 year, before the beginning of the SARS Cov19 pandemic.

All the patients signed a written informed consent for participation in a scientific study mentored by Medical University Sofia, Bulgaria (Contract number 9D/26D/2011 from Medical University Sofia). The follow-up and mortality check were conducted in accordance with the Helsinki declaration for human research, GDPR recommendations and the local Ethical committee of Medical University Sofia, Bulgaria.

### Inclusion criteria

Arterial hypertension with history of at least 1 year; left ventricular ejection fraction above 35%; antihypertensive treatment; regular self-measurement of brachial blood pressure.

### Exclusion criteria

Acute coronary syndrome with or without ST elevation and indications for urgent coronary intervention; acute stroke; acute or chronic heart failure with ejection fraction less than 35%; acute kidney dysfunction or end stage chronic kidney disease and chronic dialysis; coma, sopor or somnolence; taking antidepressants during the last 2 weeks; with impaired speech, vision, hearing; heavy head trauma; epilepsy; anemic syndrome; atrial fibrillation with suboptimal anticoagulation, verified with INR testing on enrollment; poorly controlled diabetes mellitus; hypo- or hyperglycemic coma; astenoadynamic syndrome; alcoholism; diagnosed psychiatric disease, diagnosed Alzheimer or other type of dementia.

Anamnesis and a comprehensive hypertensive history were gathered for every patient from the doctor in charge of the study. The responsible cardiologist took the physical examination. Basic laboratory testing was done as per protocol at hospitalization and included biochemistry, full blood count, liver enzymes, cardiac enzymes, lipid profile, TSH. Neuropsychological tests were conducted in private, by the certified doctor in charge of the study.

### Methods for BP measurement

#### Home-measured blood pressure (HMBP)

We taught the patients how to properly measure their blood pressure and to keep a diary for 3–7 days^22^. The target values were in compliance with current scientific recommendations^23^.

#### 24-h ambulatory blood pressure monitoring (ABPM)

Conducted with oscillometric sphygmomanometer TM2430 (Boso), validated to the requirements of the European Society of Hypertension and the British Hypertension Society. The protocol was accepted as suitable for interpretation and diagnosis if it consisted of at least 70% valid measurements, distributed proportionally during the day and night. The patients were encouraged to behave without limitation in their daily activities and to keep the regular medication regimen. Mean test duration of the recording—24 h. The cuff was put on the non-dominant hand, with measurement intervals at 15 min during the day and at 30 min during the night, with passive day and night period registration (7.00 a.m.–10.00 p.m.). Dipping index and blood pressure variability were automatically acquired.

### Neuropsychological tests used

They were conducted after full explanation of the purpose, in private, at least 1 h apart.

#### Mini mental sate examination (MMSE)^24^

The “Gold Standard” in dementia clinical screening, however, quite insensitive to the earliest phases. The cognitive domains, tested in MMSE are: orientation, registration, attention and calculation (serial 7-s or spelling backwards), recall, language and praxis (naming, repetition, 3-stage command, reading, writing, coping). We used a threshold for cognitive impairment 24 points from total of 30. The time given for completion of the test was 5–10 min.

#### Montreal cognitive assessment (MoCA)^25^

More sensitive and specific for the clinical diagnosis of the earliest stages of cognitive impairment. It is a 10-min compilation of neuropsychological tests aimed at evaluating frontal executive functioning and attention. We used the generally accepted threshold of 26 (out of maximal 30 points). Cognitive domains tested via MoCA were: attention, concentration, executive functioning, language, memory, visual-constructional, abstraction, delayed recall, orientation.

#### Geriatric Depression Scale (GDS)^26^

The coexistence of depression and dementia and the unclear association of depression with MCI led us to include this test in the initial evaluation. The test was used to study dementia patients and to explain whether it was correlated with earlier stages of cognitive impairment.

#### 4-point version of the scale for evaluating the performance in instrumental activities of daily living (4-IADL)^27^

Impaired daily functioning is art of dementia diagnosis. The test was used to better define patients with advanced cognitive impairment.

### Statistical methods

SPSS 19 was used for the statistical analysis. Descriptive analysis, *t* test, correlation analysis is used depending on the particular question. A possible association between neuropsychological tests’ results and mortality in high risk patients was assessed with logistic regression analysis. The statistic was done by a certified statistician from the National Statistical Institute of Bulgaria. The precise method, which was used depended on the means were compared, or we studied the correlation. The standard for the medical statistics 95% confidence interval was used.

### Statement of ethics

Written informed consent was obtain from all participants in the study. The study was mentored by Medical University Sofia, Bulgaria—under Contract number 9D/26D/2011 from Medical University Sofia. The study and the follow-up were conducted in accordance with the Helsinki declaration for human research and with the local Ethical committee of Medical University Sofia, Bulgaria. For the mortality rate check—ethical approval was not required as the data were fully anonymised.

### Study approval and consent to participate statements

Not applicable as this was a retrospective analysis—ethical approval was not required as the data were fully anonymized. The initial phase of neuropsychological testing and reevaluation of cardio-vascular patients was done under Contract number 9D/26D/2011 from Medical University Sofia. All initial physical examination and neuropsychological tests we done after signing informed consent.

## Results

Here we describe the follow-up results for the difference of all-cause mortality of the initially studied population. Results from the first phase have already been published^28–30^.

### Characteristics of the studied population

249 patients were included in this analysis of the mortality rate (for whom we had complete medical and passport data). During the follow-up 64 patients (25.7%) died, out of whom with MCI (MoCA < 26) were 53 (82.81%). The sex distribution was: 174 (69.9%) females and 75 (30.1%) males. The mean age was 67.83 ± 8.93 years.

All the patients had arterial hypertension (100%). The majority had also a variety of other cardio-vascular risk factors. The valid percent for high and very high cardio-vascular risk as assessed with SCORE was 19.7% (49 patients) for the high-risk group and 48.2% (120 patients) for the very high-risk group. Twenty-four—24 (9.6%) had proven stroke; 23 (9.2%) had suffered myocardial infarction and 61 (24.5%) had proven coronary artery disease. Low or intermediate kidney disfunction had 26 (10.4%). The diabetic patients were 74 (29.7%).

The comparison between dead and alive patients in terms of cognitive markers and cardio-vascular risk factors is given on Table 1.

**Table 1:** Descriptive statistic of the followed population and comparison between the mean values for deceased and for the alive.

Dead 1/alive 0	N	Mean ± SD		p (CI)
**Age at inclusion (years)**				
1	64	71.33	8.57	
0	185	66.62	8.75	< 0.001 (2.22 to 7.19)
**MoCA initial (points)**				
1	64	22.70	3.79	
0	185	24.61	3.17	< 0.001 (− 2.95 to −0.85)
**MMSE initial (points)**				
1	64	26.63	2.80	
0	185	27.99	1.79	< 0.001 (− 2.11 to −0.63)
**MoCA reevaluation (p)**				
1	64	21.52	4.16	
0	185	24.23	3.49	< 0.001 (− 3.87 to −1.56)
**MMSE reevaluation (p)**				
1	64	25.77	3.02	
0	182	27.47	2.16	< 0.001 (− 2.52 to −0.89)
**Day SBP initial (mmHg)**				
1	29	142.78	21.31	
0	97	136.15	18.80	NS
**DaySBP reev. (mmHg)**				
1	42	138.76	21.54	
0	134	136.24	17.39	NS
**Day DBP initial (mmHg)**				
1	29	78.30	9.57	
0	98	77.48	10.07	NS
**Day DBP reev. (mmHg)**				
1	42	77.96	9.03	
0	135	78.56	9.17	NS
**DayPP initial (mmHg)**				
1	29	64.45	13.56	
0	96	58.54	12.34	0.029 (0.62 to 11.21)
**DayPP reev. (mmHg)**				
1	42	60.80	15.90	
0	134	57.45	11.31	NS
**NightSBP initial (mmHg)**				
1	29	134.57	22.13	
0	98	124.75	20.43	0.028 (1.10 to 18.54)
**NightSBP reev. (mmHg)**				
1	42	129.85	25.04	
0	135	135.36	46.86	NS
**NightDBP initial (mmHg)**				
1	29	70.56	7.82	
0	98	69.48	9.86	NS
**NightDBP reev. (mmHg)**				
1	42	70.70	10.74	
0	135	70.16	12.72	NS
**NightPP initial (mmHg)**				
1	29	64.08	15.63	
0	96	55.34	13.99	0.005 (2.71 to 14.77)
**NightPP reev. (mmHg)**				
1	42	59.16	17.18	
0	134	54.72	13.67	NS
**Home SBP initial (mmHg)**				
1	64	142.47	19.98	
0	185	141.14	18.03	NS
**Home DBP initial (mmHg)**				
1	64	82.89	10.94	
0	185	83.70	10.46	NS
**Home PP initial (mmHg)**				
1	64	59.27	14.37	
0	185	57.30	12.39	NS
**Home SBP reev. (mmHg)**				
1	52	137.40	21.36	
0	150	135.55	15.50	NS
**Home DBP reev. (mmHg)**				
1	52	80.10	11.44	
0	150	80.48	9.92	NS
**Home PP reev. (mmHg)**				
1	52	57.88	13.76	
0	150	55.34	11.01	NS
**HT history (years)**				
1	64	15.80	10.64	
0	185	12.13	9.58	0.011 (0.85 to 6.48)
**Creatinine (mkmol/l)**				
1	62	119.00	9.25	
0	184	97.22	25.02	0.001 (8.78 to 34.78)
**Office SBP (mmHg)**				
1	63	143.57	27.92	
0	183	144.33	21.77	NS
**Office DBP (mmHg)**				
1	63	84.37	12.59	
0	183	86.02	11.37	NS
**Office PP (mmHg)**				
1	63	59.21	19.08	
0	183	58.31	14.80	NS

**Figure 1:**
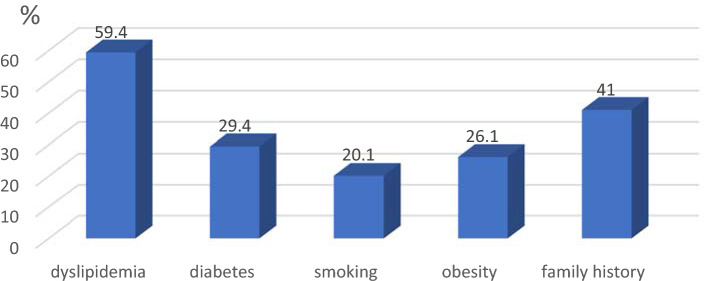
Valid percent of the risk factors in the group of patients.

The valid percent of the distribution in the various risk factor groups in shown on Fig. 1.

Twenty-three (23–9.2%) were with proven atrial fibrillation, all of whom were on anticoagulation therapy.

The distribution in the various educational levels was: university education had 106 (42.6%); high school education had 111 (44.6%); primary −31 (12.4%).

Depression had 25 (10%) of the patients. The majority 234 (94%) of the patients were autonomous. Only 10 (4%) were mildly limited in their daily activities and 2 (0.8%) were fully dependent.

All the patients were on antihypertensive medications at the time of the neuropsychological assessment. ACE inhibitors took 152 (61%); ARB—61 (24.5%); beta-blockers 182 (73.1%): diuretics 186 (74.7%); centrally acting medications 66 (26.5%); calcium channel blockers 140 (56.2%). One hundred twenty one (48.6%) were also on a statin and 186 (74.7%) on antiaggregant. Despite medical treatment 159 (63.85%) at the initial neuropsychological testing were with home blood pressure values not in the target range and 114 (56.43%—valid percent) during the reevaluation. All of the patients were on combination therapy, based on the recommended first line antihypertensive classes. As we had no fixed/recommended drug from a given class during the follow-up and all the patients were on combination therapy, it was not possible to compare death rates in patients taking certain drugs or antihypertensive classes because of overlap of effects and lack of groups on monotherapy. The study was also not designed as one for drug-related mortality reduction, we did not perform such an analysis.

### All-cause mortality rate analysis

Every patient had 2 neuropsychological tests taken at least 6 months apart. They were designated as MMSE initial and reevaluation, respectively MoCA initial and reevaluation. We assessed mortality rate 8–10 years after the second test. Sixty-four (25.7%) of the patients died for this period.

The yearly all-cause mortality rate for the last few years before the SARS Cov19 pandemic for Bulgaria was about 14.1–15.4/1000. The relative all-cause mortality rate of this studied population was about 25.7/1000 patient on yearly basis—1.67 times higher.

The relative percent of dead patients with low MoCA results (≤ 25 points) was 21.28% in comparison with only 4.42% dead in those with MoCA > 25. The distribution of the percent of the dead for MMSE is 6.83% in the group of MMSE ≤ 24 and 18.87% in those with MMSE > 24.

We used logistic regression model to analyze to what extend MMSE reevaluation and MoCA reevaluation influenced death rate in this group of patients with arteria hypertension and primarily high or very high cardio-vascular risk. The Omnibus Tests of model coefficients showed p < 0.0001, t.i. the model had some explanatory capacity. However, the Negelkerke R square was 0.146 and thus only 14.6% of the death rate could be explained by the values of the given neuropsychological test. The regression coefficient only for MoCA reevaluation was significant (p = 0.023; Exp(B) 0.864). Thus, for every point elevation of the MoCA reevaluation result, the odds that the patient was dead was elevated with 0.864, that means that it was actually lowered with 13.6%. For MMSE reevaluation the odds the patient was dead was lowered with 8.9% for every point elevation of the value of the test. We could not comment on the MMSE mortality rate as there was no significant difference if the P-value is 0.324 (Exp(B) 0.911). Much larger group should be analyzed to properly define if there was significance.

For further analysis we controlled for confounders such as: age, blood pressure variables, smoking, diabetes, peripheral artery disease, stroke, myocardial infarction, atrial fibrillation, chronic kidney disease, creatinine values, obesity, IADL, dyslipidemia, neuropsychological tests’ results. The explained variation in the dependent variable (death rate) based on our model was 35.5% (Nagelkerke *R*^*2*^). We used Forward LR binary logistic regression with dependent variable mortality for the selected time period. The CI for exp(B) was 95%, probability for stepwise entry was 0.05 and for removal—0.10. The percentage accuracy in classification was 79.7%. The variables, that remained in the equation at step 6 were MoCA reevaluation p = 0.002, B = − 0.184, Exp(B) 0.832, CI 0.741–0.934 (higher MoCA points had a protective effect); MMSE initial p = 0.049, B = − 0.201, Exp(B) 0.818, CI 0.670–0.999 (higher MMSE points had a protective effect); atrial fibrillation frequency p = 0.032, B = 1.123, Exp(B) 3.073, CI 1.104–8.553 (risk factor); diastolic blood pressure p = 0.048, B = − 0.032; Exp(B) 0.969, CI 0.939–1.000 (higher values had mild protective effect in this age group).

## Discussion

The results support the thesis, that lower results from neuropsychological tests (the more sensitive one—MoCA) and MCI in patients with arterial hypertension and high/very high cardiovascular risk lead to an elevated death rate. The mean all-cause mortality (25.7/1000) in the group as a whole is higher than the mean for Bulgaria (1.67 times higher), for the region and for Europe (10.8/1000). An important result is that 82% of the deceased in our studied group were with MCI. The regression analysis showed that 14.6% of the death rate can be explained by MCI (assessed with MoCA), that leads us to the conclusion that the cumulative negative effect of MCI and arterial hypertension may be very high with respect to mortality rate. A study of Contador et al.^31^ found that dementia mortality, but not cardio-vascular mortality, increased in patients for a period of 5 years follow-up. It was difficult to compare our results to this, as we do not have clear mortality rate by subtypes. So we lack the distinction between the reasons for mortality in our MCI patients. Another study of 2619 patients with cardiovascular risk factors^32^ found that MCI was associated with significant comorbidity, which impairs patients’ compliance and adherence to treatment, and thus the quality of cardiovascular treatment, which may be correlated with elevated death rate. A study of people above age of 60 from the National Health and Nutrition Examination Survey^33^ found that cognitive impairment with concomitant systemic vascular disease increased the risk of all-cause and cardio-vascular mortality. We found two studies to back the hypothesis for the cumulative effect of arterial hypertension and MCI (triple correlation)—the Kangwha Cohort Study^34^ and Gerontological Regional Database cohort study^35^. The second one, also with the finding that low SBP in elderly above 85 years was associated with increased mortality risk, with the possible explanation – hypoperfusion.

Several possible explanations can be given for the observed association between MCI and high cardio-vascular risk profile and hypertension. Reduced compliance to treatment, accelerated vascular impairment and impairment of the central autoregulatory mechanisms of cerebral perfusion, blood pressure, neurohormonal and vascular tone maintenance. All these mechanisms have been proven in models of hypertension and of dementia. However, their simultaneous action has been understudied in pathophysiological models. If they were proven both preclinically, clinically and on population level, that can lead to better surveillance of patients with MCI and hypertension—a screening for hypertension in MCI patients and vice-versa for timely prophylaxis.

There was not any statistically significant difference between the mean blood pressure values, cardio-vascular risk factors and diseases between the deceased and the alive patients, except for hypertension history (higher in the dead), serum creatinine (higher for the dead), pulse pressure (higher in the dead), age and neuropsychological tests’ results. The results from MoCA and MMSE were lower in the patients who were registered dead in the following years. The logistic regression analysis showed that every single point elevation in MoCA leads to 13.6% lower death rate. Such results are being reported for the first time for an Eastern European population with high cardio-vascular risk.

It should be noted that women were more than men in the group. There were several reasons for this. Women were relatively more willing to participate in such studies. Also, the relative percent females above age 65 years was relatively higher than males^36^. There was not any significant difference between the two sexes in terms of cardio-vascular disease or MCI.

These results show that 14.6% of the death rate can be explained by the results from the neuropsychological test (MoCA). This percent, however low in general, is relatively high if we consider that the group consists of patients with primarily high and very high cardio-vascular risk. In a study of Stewart et al.^37^ found that in patients with stable coronary heart disease, cognitive performance was associated with modifiable risk factors, educational status and ethnical region.

Discussing the relative percent dead in those with MCI we should bear in mind the following commentaries: The correlation between MMSE and MoCA is very high positive (Pearson r > 0.8, p < 0.001). Thus this discrepancy can be attributed to the very high MMSE threshold used (24), and to the low sensitivity of the test^38^. With lower threshold of MMSE, the death rate distribution is in accord with the general tendency, registered with MoCA. However, our aim was to show the real-world results, strengths and disadvantages of the neuropsychological tests in high-risk cardio-vascular patients, the way we see them in the everyday practice. Recently there have been papers, that review the diagnostic thresholds of MMSE and MoCA. This can lead to significant change and clarification of the general mortality rate^39^. However, we stuck to the initial official recommendations^40^, as these new and different thresholds are only in isolated studies.

## Conclusion

It is known that MCI is correlated with cardiovascular risk factors and primarily arterial hypertension. We found that the combination of MCI and AH can lead to higher mortality rate. This has important clinical implications for primary prophylaxis of cognitive decline and primary secondary prophylaxis in high risk patients. We will study further this issue to define in detail the factors responsible for the higher death rate in this group.

## Limitations

It could have been interesting to find the rate of progression for the given time of 8–10 years of patients with MCI at the first two neuropsychological assessment events. We plan to conduct a second reevaluation with neuropsychological testing and ambulatory blood pressure monitoring in the upcoming months.

## Data Availability

The datasets generated during and analyzed during the current study are not publicly available due to initial authors’ decision, but are available from the corresponding author on reasonable request in anonymised version and after discussion with all the authors.
